# Association of serum metabolome profile with the risk of breast cancer in participants of the HUNT2 study

**DOI:** 10.3389/fonc.2023.1116806

**Published:** 2023-03-16

**Authors:** Katarzyna Mrowiec, Agata Kurczyk, Karol Jelonek, Julia Debik, Guro F. Giskeødegård, Tone F. Bathen, Piotr Widłak

**Affiliations:** ^1^ Center for Translational Research and Molecular Biology of Cancer, Maria Skłodowska-Curie National Research Institute of Oncology, Gliwice Branch, Gliwice, Poland; ^2^ Department of Circulation and Medical Imaging, Norwegian University of Science and Technology (NTNU), Trondheim, Norway; ^3^ K.G. Jebsen Center for Genetic Epidemiology, Department of Public Health and Nursing, Norwegian University of Science and Technology (NTNU), Trondheim, Norway; ^4^ Clinic of Surgery, St. Olavs University Hospital, Trondheim, Norway; ^5^ Department of Medical Imaging and Nuclear Medicine, St. Olavs University Hospital, Trondheim, Norway; ^6^ Clinical Research Support Centre, Medical University of Gdańsk, Gdańsk, Poland

**Keywords:** breast cancer, cancer risk, serum metabolome, metabolic profiling, population study

## Abstract

**Background:**

The serum metabolome is a potential source of molecular biomarkers associated with the risk of breast cancer. Here we aimed to analyze metabolites present in pre-diagnostic serum samples collected from healthy women participating in the Norwegian Trøndelag Health Study (HUNT2 study) for whom long-term information about developing breast cancer was available.

**Methods:**

Women participating in the HUNT2 study who developed breast cancer within a 15-year follow-up period (BC cases) and age-matched women who stayed breast cancer-free were selected (*n*=453 case-control pairs). Using a high-resolution mass spectrometry approach 284 compounds were quantitatively analyzed, including 30 amino acids and biogenic amines, hexoses, and 253 lipids (acylcarnitines, glycerides, phosphatidylcholines, sphingolipids, and cholesteryl esters).

**Results:**

Age was a major confounding factor responsible for a large heterogeneity in the dataset, hence age-defined subgroups were analyzed separately. The largest number of metabolites whose serum levels differentiated BC cases and controls (82 compounds) were observed in the subgroup of younger women (<45 years old). Noteworthy, increased levels of glycerides, phosphatidylcholines, and sphingolipids were associated with reduced risk of cancer in younger and middle-aged women (≤64 years old). On the other hand, increased levels of serum lipids were associated with an enhanced risk of breast cancer in older women (>64 years old). Moreover, several metabolites could be detected whose serum levels were different between BC cases diagnosed earlier (<5 years) and later (>10 years) after sample collecting, yet these compounds were also correlated with the age of participants. Current results were coherent with the results of the NMR-based metabolomics study performed in the cohort of HUNT2 participants, where increased serum levels of VLDL subfractions were associated with reduced risk of breast cancer in premenopausal women.

**Conclusions:**

Changes in metabolite levels detected in pre-diagnostic serum samples, which reflected an impaired lipid and amino acid metabolism, were associated with long-term risk of breast cancer in an age-dependent manner.

## 1 Introduction

Breast cancer (BC) is currently the most commonly diagnosed malignancy with the highest number of cancer-related deaths among women. In 2020, there were over 2 million new cases and approximately 685,000 deaths due to BC worldwide; by 2040, both numbers are expected to increase by 40-50 percent (1). Primary prevention by limiting exposure to risk factors is the most effective measure to reduce the number of BC cases, which includes maintaining a healthy lifestyle through a proper weight, diet, physical activity, and avoiding smoking and drinking alcohol (2). Secondary prevention, i.e., early detection of the disease, further reduces mortality associated with BC. To increase the chances of successful treatment, BC screening programs were introduced in the 1980s and 1990s in several developed countries (2). Imaging tests, such as mammography, ultrasound, and magnetic resonance imaging, are primarily used, which, however, have some disadvantages. For example, mammography, the gold standard in BC screening gives a high percentage of false-positive results and is less reliable for the so-called dense breasts (3, 4). Therefore, there is a necessity to find the least invasive method with high sensitivity and specificity that would enable the detection of BC even before the pathological changes are visible in the biomedical images.

New opportunities for cancer diagnostics are created by metabolomics - a science that involves studying the most dynamically changing system in the human body - the metabolome. Nuclear magnetic resonance (NMR) spectroscopy and mass spectrometry (MS) are the two analytical techniques most often used in metabolomics. The analysis of metabolites in blood and other biological samples allows the detection of even subtle changes related to the development of a disease, including BC (5–9). Several studies reported that serum/plasma metabolome profiling could be used to provide signatures associated with a risk of BC. A few nested case-control studies compared pre-diagnostic samples collected from women who were cancer-free at the baseline, then developed breast cancer during a few years of follow-up, and matched women who remained healthy. These studies indicate that an increased risk of breast cancer was associated with an increased level of certain amino acids (10–12) or sex steroid metabolites (especially for postmenopausal women) (13, 14). On the other hand, an inverse association between the risk of BC and serum/plasma level of phosphatidylcholines (15), lysophosphatidylcholines (11, 16), cholesterol esters (12), and other lipid compounds (10, 12, 14) was observed. However, the importance of individual metabolites differed between studies and a common metabolic signature of the risk of breast cancer is yet to be proposed.

Recently, our group published the results of a study on risk assessment of breast cancer in the Norwegian population of women using an NMR-based metabolomics approach (17). The serum metabolites and lipids analysis included a large cohort of healthy women (more than 2400 individuals) from the Norwegian Trøndelag Health Study (HUNT2 study). The analysis enabled the detection of serum lipoprotein subfractions whose levels differentiated participants who were diagnosed with BC several years after the blood collection and those who remained free of BC during the same follow-up. Noteworthy, observed inverse associations between several very-low-density lipoprotein (VLDL) subfractions and breast cancer risk were observed primarily in the case of pre-menopausal women. In the current study we aimed to validate this observation and perform an in-depth analysis of the lipid components of serum using a targeted approach based on high-resolution MS. Current analysis included pre-diagnostic material from a group of 453 healthy participants of the HUNT2 study who were diagnosed with BC during the 15-year observation time and 453 matched women who remained cancer-free during this follow-up, which allowed for detection of components putatively associated with a risk of breast cancer.

## 2 Materials and methods

### 2.1 Study subjects

The material used in our study came from the second part of the Norwegian Trøndelag Health Study where about 65,000 healthy participants were included [the HUNT2 study (18)]; serum samples were collected between 1995 and 1997 and stored at -80°C until analysis. The matching of the HUNT2 participants to the Norwegian Cancer Registry performed in 2019 allowed the identification of 1208 women who developed breast cancer within a 22-year follow-up period (the average time to cancer diagnosis was 11.7 years). For each breast cancer case (BC), a participant that remained breast cancer-free during the whole follow-up was randomly selected as a control (Ctr), matched for age at inclusion into HUNT2 (17). For the current analysis, the above set was first split into subgroups of women who were diagnosed with breast cancer during follow-up within one-year intervals after inclusion (together with their matched controls), then 30 (or 31) case-control pairs were randomly chosen from each one-year interval from 0-1 year to 14-15 years. This ensured that the number of case-control pairs increased proportionally with the time to diagnosis. All HUNT2 participants have completed a written informed consent form, and the study was approved by the Ethics Committee of Central Norway (REK numbers #1995/8395 and #2017/2231). The baseline characteristics of 453 cases and 453 controls included in the study are shown in **Table 1**.

**Table 1:** Baseline characteristics for the study cohort.

Variable	Breast cancer (BC) cases (n = 453)	Matched controls (Ctr) (n = 453)	P-value^a^
**Age at blood collection** (years)	55.7 [13.9]	55.7 [14.0]	0.97
**Age at first menstrual period** (years)	13.5 [1.4]	13.4 [1.4]	0.64
*Missing data*	*16 (3.5%)*	*37 (8.2%)*	
**Number of full-term pregnancies** (n)			0.004*
0	44 (9.7%)	30 (6.6%)	
1	47 (10.4%)	44 (9.7%)	
2	158 (34.9%)	143 (31.6%)	
3	125 (27.6%)	123 (27.2%)	
≥ 4	78 (17.2%)	106 (23.4%)	
*Missing data*	*1 (0.2%)*	*7 (1.5%)*	
**Age at first full-term pregnancy** (years)	24.0 [4.6]	23.5 [4.0]	0.09
*Missing data*	*47 (10.4%)*	*41 (9.1%)*	
**Age at last full-term pregnancy** (years)	30.7 [5.1]	30.9 [5.0]	0.55
*Missing data*	*88 (19.4%)*	*84 (18.5%)*	
**Family history of cancer (mother)** (n)	63 (13.9%)	55 (12.1%)	0.43
**Hormone replacement therapy** ^b^ (n)			0.004*
Systemic use	75 (16.6%)	38 (8.4%)	
Local use	14 (3.1%)	8 (1.8%)	
Previous use	25 (5.5%)	31 (6.8%)	
Never use	248 (54.7%)	267 (58.9%)	
*Missing data*	*91 (20.9%)*	*109 (24.1%)*	
**Menopausal age** ^c^ (years)	48.2 [5.3]	47.0 [5.6]	0.14
*Missing data*	*355 (78.4%)*	*352 (77.7%)*	
**BMI** (kg/m^2^)	27.0 [4.8]	26.7 [4.6]	0.29
*Missing data*	*4 (0.9%)*	*3 (0.7%)*	
**WHR**	0.80 [0.06]	0.80 [0.06]	0.55
*Missing data*	*2 (0.4%)*	*1 (0.2%)*	
**Fasting status at blood collection** (time since last meal)			0.62
< 3 h	311 (68.7%)	308 (68.0%)	
3-6 h	118 (26.0%)	307 (26.0%)	
> 6 h	21 (4.6%)	26 (5.7%)	
Unknown	3 (0.7%)	4 (0.9%)	

Values are reported as mean with standard deviation [S.D.]. BMI, Body mass index; WHR, Waist-to-hip ratio. ^a^ P-value for the comparison between breast cancer cases and controls using Student’s t-test for continuous variables or Pearson’s chi-square test for categorical variables; ^b^ Current use of systemic estrogen in the form of tablet or patches; ^c^ Missing values include women that have not reached menopause and women that did not answer; * Implies significance (P-value < 0.05).

### 2.2 LC-MS targeted metabolomics

The Absolute IDQ p400 HR kit (test plates in the 96-well format; Biocrates Life Sciences AG, Innsbruck, Austria) was used to perform targeted quantitative analysis of the metabolites, according to the manufacturer’s protocol. The kit allows the quantitative measurement of up to 408 compounds (or their isomer groups) covering 11 classes of metabolites (including amino acids, biogenic amines, hexoses, acylcarnitines, di- and triglycerides, (lyso)phosphatidylcholines, sphingolipids, and cholesteryl esters) in 10 µl of sample thanks to the combination of direct flow injection and liquid chromatography (LC) with mass spectrometry (MS). The MS analyses were performed using Orbitrap Q Exactive Plus spectrometer (Thermo Fisher Scientific, Waltham, MA, USA), which was equipped with a 1290 Infinity UHPLC (Agilent, Santa Clara, CA, USA) system; Xcalibur 4.1 software (Thermo Fisher Scientific, Waltham, MA, USA) controlled the whole system. The concentrations of metabolites in µM were established by processing the registered spectra and chromatograms using Xcalibur 4.1. and MetIDQ DB110-2976 software (Biocrates Life Sciences AG) (19).

### 2.3 Data processing

The metabolomics dataset consisted of 906 samples, each described by measured levels of 389 metabolites. Two types of missing values were detected in the dataset: (i) values below the limit of detection (i.e., missing not at random), and (ii) measurements lacking due to the internal standard error (i.e., missing completely at random). Fifty and ten percent of missing values in all samples were allowed for either type of error, respectively, as recommended by (20); otherwise, the compound was excluded from further analyses. The final dataset qualified for quantitative analysis comprised 284 metabolites; for 105 metabolites the thresholds for the acceptable number of missing values were exceeded. The final dataset of 284 metabolites was batch-corrected using an empirical Bayes method (21). We assumed that samples measured using one 96-well sample preparation plate represent one batch. Before batch adjustment, data were transformed using the log base 2 function. The batch-corrected data were processed for missing values imputation. Values below the limit of detection were replaced with random numbers generated from normal distribution truncated to a segment between 0 and the median value of the limits of quantitation for all test plates. Other lacking measurements (missing completely at random) were filled by values imputed using the k-nearest neighbor approach; the nearest observed data were identified using a correlation distance metric, and the mean value of the three nearest neighbors was used [based on measurements collected for the same group (cases or controls)].

### 2.4 Statistical and bioinformatics analyses

The difference in baseline characteristics between future breast cancer patients and controls was tested with Student’s t-test for continuous variables and Pearson’s chi-square test for categorical variables. The Mann-Whitney U test was applied to test the statistical significance of differences between the analyzed groups of individuals in measured levels of 284 compounds qualified for quantitative analysis. The Benjamini-Hochberg procedure was applied to minimize the number of false positive results. All statistical hypotheses were tested at the 5% significance level. Moreover, following the Mann-Whitney U test, the “r” effect size was calculated according to the formula: $r=\frac{z}{\sqrt[{2}]{N}}$ (where z is the value of the test statistic and N is the total number of observations in two compared groups) with interpretation according to the Cohen’s criterion (r ≤ 0.1 represents negligible effect size) (22). The chi-square independence test was applied for the remaining set of 105 metabolites not qualified for quantitative analysis to test whether a given compound’s absence/presence status was a group-related feature. In addition, Spearman’s rank correlation coefficients were calculated to determine the degree of association between the levels of metabolites and the age at inclusion or time of cancer diagnosis. Three age-related subgroups of participants (i.e., younger, average, older) were identified using the Gaussian mixture model. Odds ratios (OR) and 95% Wald confidence intervals (CI) were calculated for one standard deviation (SD) increase in the concentration of each variable using unconditional logistic regression; the logistic regression models were fitted separately for three age-related subgroups of women. Multivariate predictive models were fit using partial-least squares discriminant analysis (PLS-DA) for discriminating between cases and controls in age-related subgroups of women. The number of latent variables (LVs) giving the minimum cross-validated test error (inner loop) was chosen; the models were validated using double tenfold cross-validation with 30% of the samples included in the test sets of the inner and outer loops, and their significance (Pperm ≤ 0.05) was assessed by permutation testing (1000 permutations). The metabolic pathway enrichment analysis was performed using the MetaboAnalyst 5.0 platform for all quantitative data (https://www.metaboanalyst.ca/MetaboAnalyst/ModuleView.xhtml, accessed on 26 August 2022).

## 3 Results

Metabolite profiles were analyzed by a mass spectrometry-based approach in a set of 906 serum samples collected from healthy women participating in the HUNT2 population-based study. The set included 453 women who developed breast cancer within a follow-up period and 453 age-matched women who remained free of breast cancer during the follow-up (BC and Ctr, respectively, afterward). Baseline characteristics for the study cohort are presented in **Table 1**. According to provided data, two known epidemiological factors were associated with breast cancer risk in the study cohort: multiple pregnancies were more frequent among controls (p=0.004) while systemic use of hormone replacement therapy (HRT) was more frequent among BC cases (p=0.004). There were 389 metabolites detected, among which 284 compounds were quantified in the majority of samples and used in quantitative analyses, including 30 amino acids and biogenic amines, one sugar (hexose), and 253 lipids or their isotope groups (32 acylcarnitines, 56 glycerides, 119 glycerophospholipids, 32 sphingolipids, and 14 cholesteryl esters). When differences in serum concentrations of quantified compounds were assessed between both major groups in total, no statistically significant differences remained after multiple testing corrections; similarly, the effect size of differences was negligible for all compounds.

### 3.1 Levels of serum metabolites correlate with the participant’s age

**Figure 1:**
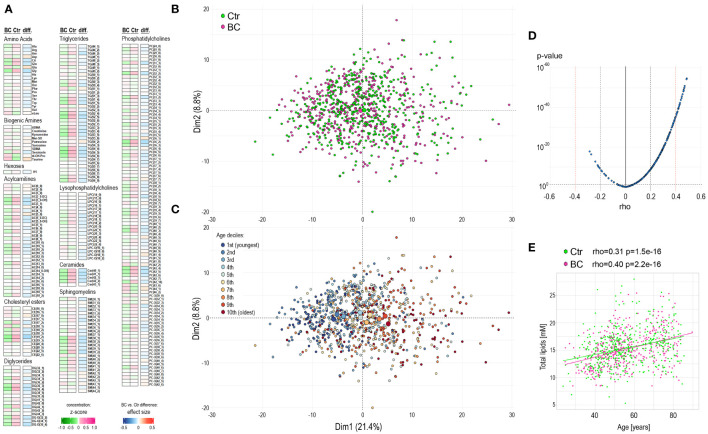
Characterization of the serum metabolome profile in participants of the HUNT2 study. **(A)** – Levels of metabolites in pre-diagnostic serum samples from 453 participants who were diagnosed with breast cancer during 15-year follow-up (BC) and 453 age-matched women who remained cancer-free during this time (Ctr); heatmap represents average levels of analyzed metabolites in each group (represented as z-score) and significance of differences between groups (represented as “r” effect size). **(B)** – Principal component analysis (PCA) of general similarities between samples; samples of women diagnosed with cancer or remaining cancer-free are marked separately (large circles represent the average for each group). **(C)** – PCA in a total of 906 samples with subsequent deciles of participant ages marked separately. **(D)** – the significance of the correlation between serum metabolite concentrations and the age of study participants (all participants together). **(E)** – the correlation of aggregated concentration of all analyzed lipids and age of study participants (controls and BC cases are marked separately).

The mean concentrations of quantified compounds and the strength of differences between the groups of participants are presented in **Figure 1A** (and specified in **Supplementary Table S1**). Moreover, the remaining set of 105 metabolites not qualified for quantitative analysis tested for the absence/presence status did not show any differences between the groups (likewise, such differences were not detected in any other comparisons performed further; not shown). Unsupervised principal component analysis (PCA) of all 906 samples showed a large heterogeneity (**Figure 1B**), with no clear clusters according to the BC and Ctr groups. Interestingly, we found that the age of participants was the major factor responsible for observed variation, which is depicted in **Figure 1C**. Subsequently, we noted a strong correlation between the participant’s age and concentrations of serum metabolites (**Figure 1D**), which was irrespective of the BC or Ctr statuses (**Supplementary Table S2** and **Supplementary Figure S1**). This correlation is exemplified by the aggregated concentration of serum lipids – increased total lipid concentrations were generally observed in older women (**Figure 1E**); importantly, only the aggregated level of lysophosphatidylcholines (LPC) did not correlate with age when major classes of lipids were considered.

### 3.2 The serum metabolome profiles differ for participants that later develop breast cancer and controls

**Figure 2:**
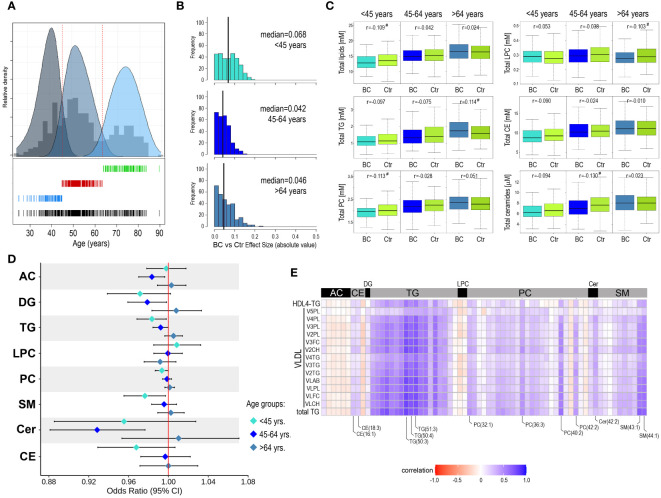
Metabolome profiles in serum of younger and older participants of the HUNT2 study. **(A)** – Distribution of age of participants selected for the analysis (all participants together); three subgroups were identified by the components of the Gaussian mixture model: <45-year-old, 45-64-year-old, and >64-year-old (bottom). **(B)** – Distribution of metabolites that showed the increased significance of differences between BC cases and controls in three age-defined subgroups of participants (vertical lines represent the median of effect sizes). **(C)** - Differences in aggregated concentration of different classes of lipids between BC cases and controls (in three age-defined subgroups of participants); hashtags marked at least a small effect size. **(D)** – Odds ratios (and 95% confidence intervals) for the association between increased concentration of selected metabolites and risk of breast cancer during follow-up in age-defined subgroups. **(E)** – The correlation between the concentration of serum lipids measured by HR-MS (current study) and levels of NMR-measured lipoprotein particles (Debik et al., 2021) in the subgroup of younger participants (<45-year-old); showed lipids with at least a small effect size of differences between BC and Ctr groups (r>0.1) and lipoprotein particles with adjusted p-value <0.05 (AC – acylcarnitines, DG – diglycerides, CE – cholesteryl esters, Cer – ceramides, FC – free cholesterol, LPC – lysophosphatidylcholines, PC – phosphatidylcholines, PL – phospholipids, SM – sphingomyelins, TG – triglycerides).

Considering the strong correlations between metabolite concentrations and participants’ age, we analyzed differences between the BC and Ctr groups in smaller subgroups to limit age-related variance. We assumed that three age-defined subgroups could be hypothetically distinguished: “younger than average”, “average/middle-aged”, and “older than average”. Based on the actual age distribution in the study cohort these three subgroups were identified as components of the Gaussian mixture model: below 45-year-old (112 BC/Ctr pairs), 45-64-year-old (206 BC/Ctr pairs), and above 64-year-old (135 BC/Ctr pairs), which is depicted in **Figure 2A**. After such a split of the study cohort, several compounds with statistically significant differences between the BC cases and controls were detected. In general, for 123 compounds effect size was higher than negligible (r>0.1) in either age-defined subgroup (**Supplementary Table S3**). It is noteworthy, that the largest differences between the BC cases and controls were noted for younger women (< 45 years old): 82 compounds with r>0.1 (median of effect sizes equal to 0.068). For the two remaining subgroups, there were 16 and 52 differentiating compounds (median of effect sizes equal to 0.042 and 0.046) for the 45-64-year-old and > 64-year-old, respectively (**Figure 2B**). Differences between the BC cases and controls in three age-defined subgroups are depicted for aggregated concentrations of major classes of serum lipids in **Figure 2C**. In general, except for LPC, lipids concentrations increased with age, however, differences between the BC cases and controls were more heterogenous among age-defined subgroups. For younger and middle-aged women, lipids concentrations were generally lower in the BC cases (except LPC). Interestingly, in contrast to phosphatidylcholines (PC), concentrations of LPC increased in younger women (<45 yrs) who eventually developed breast cancer during follow-up, which resulted in a higher overall LPC to PC ratio. On the other hand, for older women (>64 yrs.), lipids concentrations (particularly glycerides) were generally higher among the BC cases.

### 3.3 In younger women, higher concentrations of serum lipids reduce the risk of breast cancer

To further assess the association between the risk of breast cancer and the concentration of serum metabolites, odds ratios (with 95% confidence intervals) were estimated for all 284 compounds in each age-defined subgroup separately. Though a few specific metabolites showed some association with cancer risk, it was generally weak in all age-defined subgroups (**Supplementary Figure S2**). Further, odds ratios were estimated for aggregated concentrations of major classes of serum lipids (**Figure 2D**). We observed that in subgroups of younger and middle-aged women, increased concentrations of glycerides (both DG and TG), phosphatidylcholines, and sphingolipids were generally associated with a reduced risk of cancer. On the other hand, increased concentrations of lipids (except LPC) with the highest increases in glycerides and ceramides, were associated with an increased risk of cancer in the subgroup of older women. However, only a few of these associations appeared statistically significant: TG and SM in the <45-year-old subgroup while DG, TG, and Cer in the 45-64-year-old subgroup. Moreover, we applied partial-least squares discriminant analysis (PLS-DA) for discriminating between BC cases and controls in age-defined subgroups of women (**Supplementary Figure S3**). This analysis showed low prediction accuracy of the classification models, which was in the range between 52% and 55% (highest in the group of older women), and only the model for the older women was significant (Pperm<0.05).

### 3.4 Lipids whose levels are associated with breast cancer risk correlate with lipoprotein subfractions

We observed that differences in serum lipid concentrations between BC cases and controls were more frequent in the subgroup of younger women (below 45 years old), which were putatively pre-menopausal (actual menopause status was missing for the majority of study participants). Hence, this observation was coherent with our previous results of the NMR-based metabolomics study performed in the cohort of HUNT2 study participants (17). In that study, the inverse correlation between the level of several VLDL subfractions and triglycerides (total serum level and HDL4 TG) and the long-term risk of breast cancer was observed for pre-menopausal women (actual status or age below 51 years). Therefore, to deepen knowledge about this effect, data regarding levels of NMR-detected lipoproteins were retrieved for all 906 women included in the present study. The general correlations between all NMR-assessed lipid particles and MS-assessed lipids species are depicted in **Supplementary Figure S4**. As one could expect, we observed a strong positive correlation between concentrations of the majority of serum glycerides (both DG and TG) and concentrations of VLDL, intermediate-density lipoproteins (IDL), and triglyceride-containing high-density lipoproteins (HDL-TG) We also found positive correlations between concentrations of some cholesteryl esters (e.g., CE(16:1) and CE(18:3) and acyl-alkyl-phosphatidylcholines (A-AG-phosphocholines) and levels of low-density lipoproteins (LDL) and HDL. Moreover, several sphingolipids (both SM and ceramides) correlated with levels of VLDL, LDL, and HDL-TG particles. In the next step, we searched for the correlation between concentrations of selected serum lipids that showed significant differences between BC cases and controls in the subgroup of younger women (<45-year-old, at least small effect size) and levels of lipoprotein subfractions, mostly VLDL ones, associated with risk of breast cancer in the subgroup of pre-menopause women according to Debik et al. (17), which is shown in **Figure 2E**. In general, strong associations were observed between discriminatory lipoprotein subfractions and discriminatory triglycerides. However, levels of discriminatory VLDL particles also correlated positively with concentrations of certain discriminatory cholesteryl esters, phosphatidylcholines, ceramides, and sphingomyelins (compounds marked in **Figure 2E**), which suggested their presence in the lipoprotein (sub)fractions of interest.

### 3.5 Earlier and later diagnoses of breast cancer correlate with serum metabolome profiles

**Figure 3:**
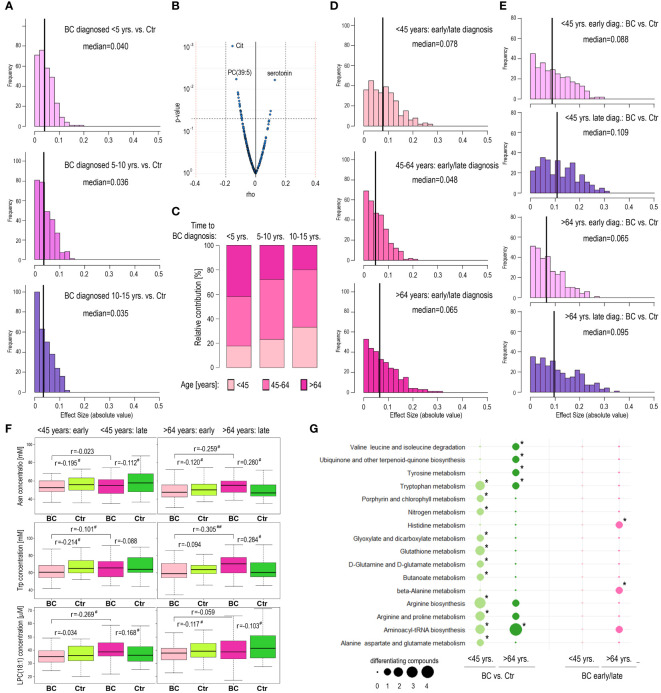
Metabolome profiles in pre-diagnostic serum samples of HUNT2 study participants with earlier and later diagnoses of breast cancer. **(A)** – Distribution of metabolites that showed the increased magnitude of differences between BC and corresponding controls in three subgroups of participants with different times to cancer diagnoses during follow-up, 5 years (early); 5-10 years; and 10-15 years (late). **(B)** – The significance of the correlation between serum metabolite concentrations in pre-diagnostic samples and time to cancer diagnosis. **(C)** – Contribution of women at different ages during inclusion (<45-year-old; 45-64-year-old; and >64-year-old) in subgroups of BC cases diagnosed during 5 years (early); 5-10 years; and 10-15 years (late) follow-up. **(D)** – Distribution of metabolites that showed the increased magnitude of differences between participants with early (<5 years) and late (10-15 years) diagnoses of breast cancer in three age-defined subgroups of participants (<45-year-old; 45-64-year-old; and >64-year-old). **(E)** – Distribution of metabolites that showed the increased magnitude of differences between BC and corresponding controls in four subgroups of participants, younger/early diagnosis; younger/late diagnosis; older/early diagnosis; and older/late diagnosis. **(F)** – Differences in concentration of selected metabolites (Asn; Trp; and LPC(18,1)) between BC cases and controls in four subgroups of participants, younger/early diagnosis; younger/late diagnosis; older/early diagnosis; and older/late diagnosis; differences in pre-diagnostic samples of participants with an earlier and later cancer diagnosis are also marked (# - small effect size; ## - medium effect size). **(G)** – Metabolic pathways associated with compounds whose levels were different between BC cases and controls in subgroups of younger and older participants (<45-year-old and >64-year-old; respectively) as well as between younger and older participants with an early and late cancer diagnosis (<5 years and 10-15 years follow-up; respectively); significantly overrepresented pathways are marked with asterisks (size of a dot corresponding to the number of differentiating compounds associated with a given pathway).

The method of selection of participants for the current study (i.e., equal distribution of BC cases diagnosed with cancer at different time intervals from sample collection matched with corresponding controls) was aimed to analyze whether time to cancer diagnosis affected molecular differences observed between BC and Ctr groups. Hence, in the next step, we compared differences between BC cases and controls in subgroups where breast cancer was diagnosed up to 5-year follow-up (“early” diagnosis), during 5 to 10 years follow-up, and during 10 to 15 years follow-up (“late” diagnosis). However, this analysis revealed similar numbers of discriminatory compounds in all three time-of-diagnosis-related subgroups. There were 16, 18, and 18 compounds with higher than negligible effect size (r>0.1) between BC and Ctr in subgroups <5, 5-10, and 10-15 years-to-diagnosis, respectively (details in **Supplementary Table S4**); corresponding medians of effect sizes were equal to 0.040, 0.036, and 0.035 (**Figure 3A**). Moreover, when a small subgroup of women who were diagnosed with cancer within one year after participation in the HUNT2 study (n=30) was analyzed, only a few metabolites showed significantly different levels compared to matched controls (3 compounds with a medium effect size r>0.3 and the median of effect sizes equal to 0.073; **Supplementary Table S5**). Nevertheless, we observed several metabolites whose concentrations in BC cases were correlated with the time of diagnosis (**Figure 3B**; **Supplementary Table S6**). Interestingly, we noted a statistically significant negative correlation between the participant’s age (at the moment of sample collection) and the time of cancer diagnosis (length of the follow-up) (**Supplementary Figure S5**). We found that in the subgroup of BC cases with early-detected cancer (i.e., <5 years follow-up) was an over-representation of older women (>64-year-old), while in the subgroup of BC cases with late-detected cancer (i.e., 10-15 years follow-up) was an over-representation of younger women (<45-years-old) (p=0.003), which is depicted in **Figure 3C**. This observation suggested that the correlation between metabolite concentrations and time of cancer diagnosis was a function of a much stronger correlation between metabolite concentrations and the age of participants (which could be exemplified by compounds with the strongest correlation with time-to-diagnosis: citrulline, PC(39:5), and serotonin). Nevertheless, this observation indicated that hypothetical differences between “early-diagnosed” and “late-diagnosed” cases should be analyzed in the age-defined subgroups separately.

### 3.6 Metabolic features of women with different time to cancer diagnoses depend on their age

We noted that the number of metabolites differentiating BC cases with early/late diagnosis was similar in the subgroups of younger and older participants: 104 and 93 metabolites with higher than negligible effect size (r>0.1), respectively; median values of effect sizes equal to 0.078 and 0.065, respectively (**Figure 3D**). However, different metabolites discriminated cases with early and late diagnosis in either age-defined subgroup (**Supplementary Table S7**). Furthermore, we searched for metabolites discriminating between BC cases and controls within different subgroups defined by age and time of cancer diagnosis (**Supplementary Table S8**). The number of metabolites discriminating BC and Ctr was higher in the case of late-diagnosed cancers than in the case of early-diagnosed cancers in either age-defined subgroup and the significance of differences was highest in the subgroup of younger women (median of effect sizes equal to 0.109; **Figure 3E**). However, different metabolites discriminated BC cases and controls in different age-defined and diagnosis-related subgroups, which is exemplified by the 3 compounds in **Figure 3F**. For example, serum concentration of asparagine (Asn) was significantly higher in late-detected BC cases compared to early-detected BC cases only in the subgroup of older women. On the other hand, the concentration of this amino acid was higher in BC samples (when compared to Ctr samples) in the case of older women with late-diagnosed cancer, while its concentration was lower in BC samples in the case of younger women with late-diagnosed cancer. To further analyze these age- and diagnosis-related differences, metabolic pathways associated with different sets of discriminatory compounds were identified. We found that some of the metabolic pathways associated with compounds differentiating BC and Ctr cases, including degradation of branched amino acids and glutathione metabolism, differed among younger (<45 yrs.) and older (>64 yrs.) women (**Figure 3G**). Overrepresentation of pathways associated with metabolites differentiating early and late-diagnosed cancer (His metabolism and Ala metabolism) was observed only in the subgroup of older women. Hence, this observation further confirmed the age-related nature of metabolic features associated with the risk of breast cancer.

## 4 Discussion

In this study, pre-diagnostic serum samples from more than 900 healthy participants of the population-based HUNT2 study were analyzed by a targeted high-resolution mass spectrometry-based approach to defining the quantitative profile of the serum metabolome. The analyzed group included women who were diagnosed with breast cancer during a 15-year observation period and age-matched women who remained breast cancer-free during this follow-up. This allowed us to search for components putatively associated with a risk of breast cancer. This work has a similar design to a few other studies performed recently using different analytical platforms and targeting different cohorts (10–17, 23, 24). A strong correlation between serum metabolite concentrations and participants’ age was observed in our study, which putatively reflected the adaptation of energy and lipid metabolism due to age-related changes in hormonal balance. Aging women experience decreased estrogen and increased androgen levels, which together with lifestyle changes affect overall energy metabolism and body fat redistribution. Enhanced lipolysis of visceral fat results in the production of excessive free fatty acids, which in the combination with reduced β-oxidation increases lipid synthesis (25, 26). Moreover, in addition to lipids and fatty acids, serum concentrations of amino acids also changed with women’s age (27). As a consequence, age was the major confounding factor contributing to a large heterogeneity in the data that affected the ability to find discriminatory components associated with higher risks of developing breast cancer. Therefore, analyses were performed in smaller subgroups of age-matched case-control pairs. Nevertheless, it is important to note that previously published reports addressed cohorts of women of different ages; with an average age ranging from 45 to 68 years. Hence, different participants’ ages (together with different analytical platforms) may contribute to possible discrepancies among published studies regarding specific metabolites.

We found that several specific metabolites showed different serum concentrations between women who eventually developed breast cancer and matched controls, yet the effect sizes of such differences were relatively low. Therefore, assuming the possible functional redundancy of compounds that belong to the same class of lipids, aggregated concentrations of lipid classes were also addressed. We found that in the group of younger and middle-aged women, particularly women below 45 years, increased levels of glycerides (diglycerides and triglycerides), phosphatidylcholines, and sphingolipids (ceramides and sphingolipids) were associated with reduced risk of cancer. This general observation was coherent with the results of previously published reports. Brantley and coworkers reported that poly-unsaturated triglycerides and cholesteryl esters were inversely associated with breast cancer in the American Nurses’ Health Study (12). His and coworkers, using an earlier version of the analytical platform applied in the current study, showed the inverse correlation of several phosphatidylcholines and sphingomyelins with breast cancer risk in the European Prospective Investigation into Cancer (EPIC) cohort (15). Notably, a similar risk association of two specific metabolites studied in-depth in that report, acylcarnitine AC(2:0) and PC(36:3), was found in our study (increased and reduced risk, respectively). Furthermore, similar to the study performed by Kühn and coworkers (16), a reduced level of LPC(18:0) was noted in women who developed breast cancer. Therefore, the association between reduced levels of serum lipids (glycerides, phosphatidylcholines, and sphingolipids in particular) and increased risk of breast cancer, at least in younger and middle-aged women, appeared as a general observation in several independent studies.

Similar to the MS-based studies mentioned above, NMR-based profiling of plasma in the French SU.VI.MAX cohort revealed that women with lower levels of glycerol-derived compounds, unsaturated lipids, and lipoproteins, had a higher risk of developing breast cancer (10). Another NMR-based profiling of pre-diagnostic serum samples was performed more recently by our group in the large group of the HUNT2 study participants (1199 case-control pairs), which revealed the inverse association of breast cancer risk with several subfractions of VLDL particles (as well as TG-containing HDL particles) specifically in the subgroup of premenopausal women (17). Using the same set of samples, we searched for correlations between levels of lipid species (analyzed here by MS) and lipoprotein particles (analyzed previously by NMR). It is known that triglycerides are major constituents of VLDL particles, cholesterol, and cholesteryl esters are major constituents of LDL particles, and cholesterols and phospholipids are major constituents of HDL particles (28). The results of our correlation analysis fit this general pattern, adding some interesting extensions like the possible enrichment of certain diglycerides, phosphatidylcholines, ceramides, and sphingomyelins in VLDL particles. Nevertheless, a strong correlation between discriminatory VLDL particles and discriminatory triglycerides (and certain phospholipids) was noted, which further confirmed the robustness of serum lipids as potential markers of breast cancer risk.

Except for lipids, serum concentration of several “small molecule” metabolites (amino acids, biogenic amines, and hexoses) was addressed in the current study, and such compounds were also associated with breast cancer risk (though the magnitude of differences established by effect size was relatively low). Nevertheless, a few amino acids showed a similar trend of association with breast cancer risk as described in previous studies. This included the association between a reduced risk of cancer and higher levels of Arg, Gln, Lys, and Thr, previously reported by others (12, 15). Moreover, when metabolic pathways associated with risk-related compounds were identified, pathways involved in the metabolism of aromatic amino acids (Phe, Tyr, Trp) and branched-chained amino acids (Val, Leu, Ile) were found both in our study and the study of Yoo and coworkers (11). Therefore, reduced serum level of specific amino acids seems a general metabolic feature associated with an increased risk of breast cancer. Notably, metabolites putatively associated with breast cancer risk are known to be key components of cancer-related metabolism in actual breast cancer. For example, glutamine metabolism (putatively associated with reduced levels of Gln and Glu in BC cases) connected to a tricarboxylic acid cycle is an important pathway supporting cancer cell growth (29). Reduced plasma/serum levels of amino acids putatively associated with cancer risk (Ala, Arg, Ile, Leu, Tyr, Trp, Val) were observed in patients with actual breast cancer (30, 31). On the other hand, increased accumulation of amino acids (and over-representation of metabolic pathways related to amino acids) is a characteristic feature of breast tumors (32). Hence, it is important to note that reduced levels of circulating amino acids could be associated with the overexpression of amino acid transporters typical for breast cancer cells (33).

Data presented in our study, in coherence with previously published reports, revealed that reduced serum levels of several lipids and amino acids were associated with an increased risk of developing breast cancer. It has been observed that reduced levels of certain metabolites in the blood of actual cancer patients could reflect the increased transfer to tumor tissue due to higher consumption of these metabolites by cancer cells (34). However, it is unlikely that the increased demand for metabolites by cancer cells contributes to the reduced concentration of these metabolites in the serum of women who developed cancer during follow-up yet were considered cancer-free at the time of blood collection. One could assume that women who were diagnosed with cancer shortly after the inclusion in the study actually might have cancer at an early pre-clinical stage, yet the impact of these hypothetical cases would be low when the whole cohort is considered. Although we found a correlation between metabolite concentrations in pre-diagnostic samples and time to breast cancer diagnosis, such a correlation putatively reflected a reverse correlation between age and time of diagnosis and a very strong association of metabolite concentration with age. Moreover, the extent of differences between BC cases and corresponding controls was similar for women who were diagnosed earlier (within 5 years of follow-up) and later (after more than 10 years of follow-up). Furthermore, the extent of such differences was comparably low even in the subgroup of women who were diagnosed with cancer within one year after sample collection (i.e., women who probably had the asymptomatic disease during sample collection). Additionally, when age-defined subgroups were analyzed separately, there were more metabolites differentiating BC and controls for late-diagnosed than early-diagnosed cases. Therefore, specific features of the serum metabolome profile detected in women who eventually developed cancer during follow-up were associated rather with cancer-promoting/enabling factors affecting their metabolism than metabolic changes related to the presence of pre-clinical/asymptomatic disease.

In general, breast cancer represents a hormone-dependent malignancy, hence conditions affecting hormonal homeostasis are among known risk factors, including reproduction-related factors (e.g., number and age of pregnancies) (35) and the usage of hormone replacement therapy (36). In our cohort, multiple full-term pregnancies were associated with a reduced risk of breast cancer while systemic usage of HRT was associated with an increased risk of cancer, which brought our attention to the metabolic changes associated with these two conditions. The usage of HRT markedly affects metabolism and the serum metabolic profile (37, 38). A newer metabolomics study showed that postmenopausal usage of estrogen (alone or in combination with progestin) affected serum concentrations of about 200 metabolites in women from the American Cancer Prevention II Nutrition Cohort. In general, levels of several amino acids, acylcarnitines, sphingolipids, phosphatidylcholines, and steroids were reduced in HRT users (triglycerides were not assessed in that study) (39). Noteworthy, among these metabolites were those whose reduced levels were associated with increased cancer risk in middle-aged and older women in our study (e.g., Glu, Gly, LPC(18:0), or AC(18:2)). On the other hand, it was reported that HRT in postmenopausal women was associated with increased serum levels of triglycerides and VLDL particles (40). However, a larger meta-analysis showed that the influence of HRT on serum levels of cholesterols, triglycerides, and lipoprotein particles depends on the combination of hormones and methods of their application (41), which indicated a more complex nature of the association between HRT and metabolism. Nevertheless, since HRT is applied in postmenopausal women, other metabolism-affecting risk-related factors should be considered in a group of younger premenopausal women. Among them is pregnancy, which requires complex physiological adaptation and reprogramming metabolism of a mother. (Multiple) full-term pregnancies, which are inversely associated with the risk of breast cancer, could participate in the metabolic characteristics of premenopausal women. Therefore, it should be mentioned that the concentration of triglycerides and cholesterol (total and lipoprotein) increases during normal pregnancy (42). A more recent metabolomic study showed increased levels of several amino acids and phospholipids (phosphatidylethanolamines and phosphatidylcholines) during pregnancy (43). Moreover, this study showed significantly reduced levels of LPCs in pregnant women, which resembled the association between generally increased levels of LPCs and enhanced risk of breast cancer. Therefore, pregnancy-related changes in metabolism might contribute to serum metabolome features observed in the group of younger women. Weight, another hormone- and metabolism-related condition, was not identified as a risk factor in the analyzed HUNT2 cohort. However, several other conditions can also be considered hypothetical factors affecting metabolism in women who will eventually develop cancer. Among general cancer-enabling/promoting features is chronic inflammation (44). Increased serum level of LPC was reported in several inflammation-related conditions (45). Hence, an increased ratio of LPC to PC observed in the serum of women who will develop cancer also fits the cancer-enabling characteristics.

In conclusion, differences in concentration of several metabolites were noted in pre-diagnostic serum samples between women who eventually developed breast cancer and women who stayed breast cancer-free. Metabolomics features detected at the baseline (i.e., during recruitment to the study) were associated with the long-term risk of breast cancer in an age-dependent manner and could be observed primarily in younger (mostly pre-menopausal) women. Changes in metabolite profiles putatively reflected the reprogramming of metabolism connected to cancer-promoting conditions, including changes in hormone homeostasis. The study indicated that impaired metabolism of lipids and amino acids is an etiological factor involved in breast cancer, that could be observed many years before cancer diagnosis. However, despite a relatively large number of participants and the direct quantitative value of the applied analytical platform, which were strengths of the study, the associations between specific metabolites and breast cancer risk were generally modest. Hence, the predictive value of a hypothetical metabolomics signature appeared too low for application in cancer-screening strategies. On the other hand, thanks to the inclusion of a relatively large and heterogenous cohort, the correlation between metabolite concentration and age has been convincingly documented and demonstrated as an important obstacle in the cancer risk biomarker-oriented study. Moreover, the extended knowledge of metabolomics profiles associated with breast cancer risk could help to develop rational prevention strategies based on modified dietary and lifestyle patterns.

## Data availability statement

The original contributions presented in the study are included in the article/**Supplementary Material**. Further inquiries can be directed to the corresponding author.

## Ethics statement

The studies involving human participants were reviewed and approved by the Ethics Committee of Central Norway. The patients/participants provided their written informed consent to participate in this study.

## Author contributions

Conceptualization: PW, GG, TB. Methodology: KM, KJ. Investigation: KM, KJ. Data curation: AK. Formal analysis: AK, JD. Writing- original draft: PW, KM, Writing- Review & Editing: KJ, JD, GG, TB. Visualization: KM, JD. Funding: PW. All authors contributed to the article and approved the submitted version.
