# Supplementary figures and images for: Association of serum metabolome profile with the risk of breast cancer in participants of the HUNT2 study
Source: Front Oncol. 2023 Mar 16;13:1116806. doi: 10.3389/fonc.2023.1116806 (PMC10061137; doi:10.3389/fonc.2023.1116806)

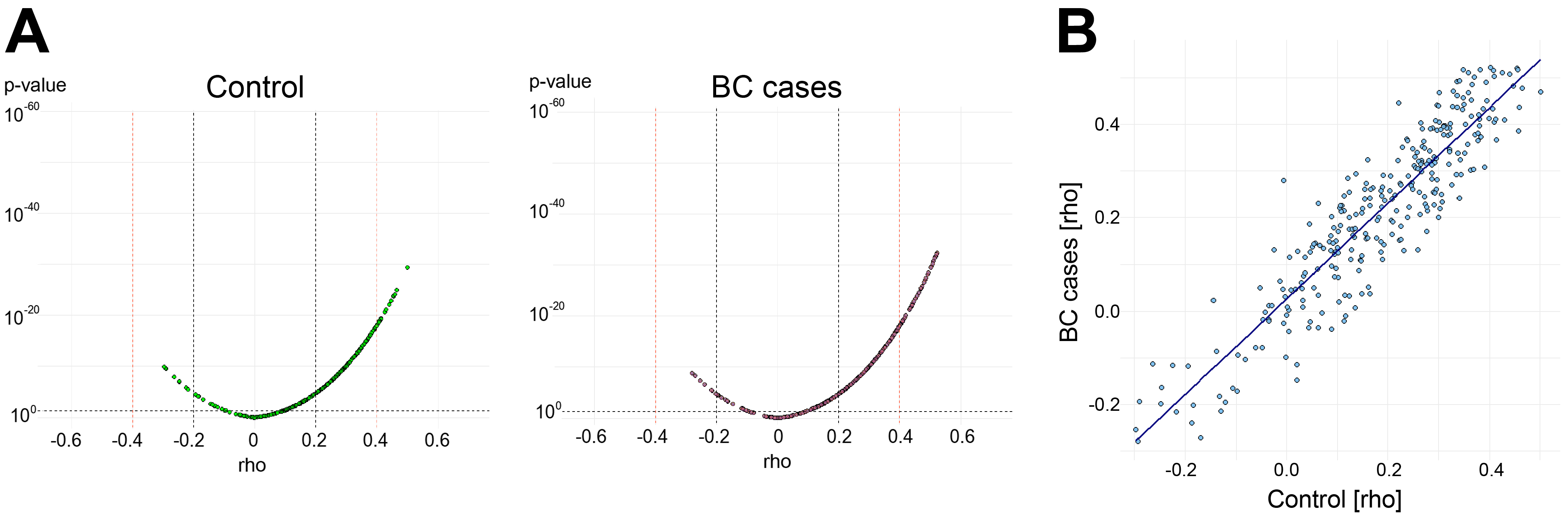

Supplement: Supplementary file 1 [file Image_1.jpeg]

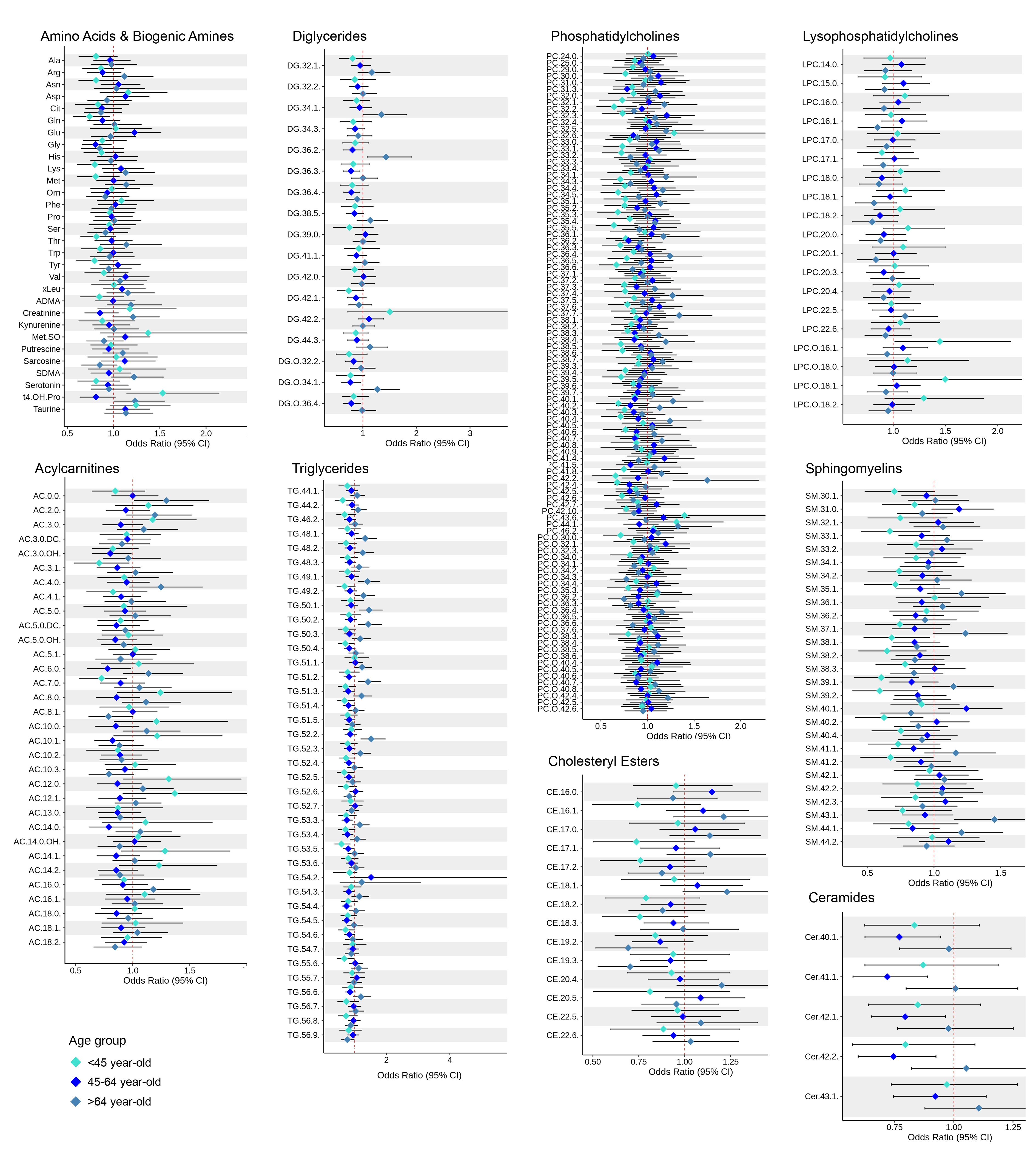

Supplement: Supplementary file 2 [file Image_2.jpeg]

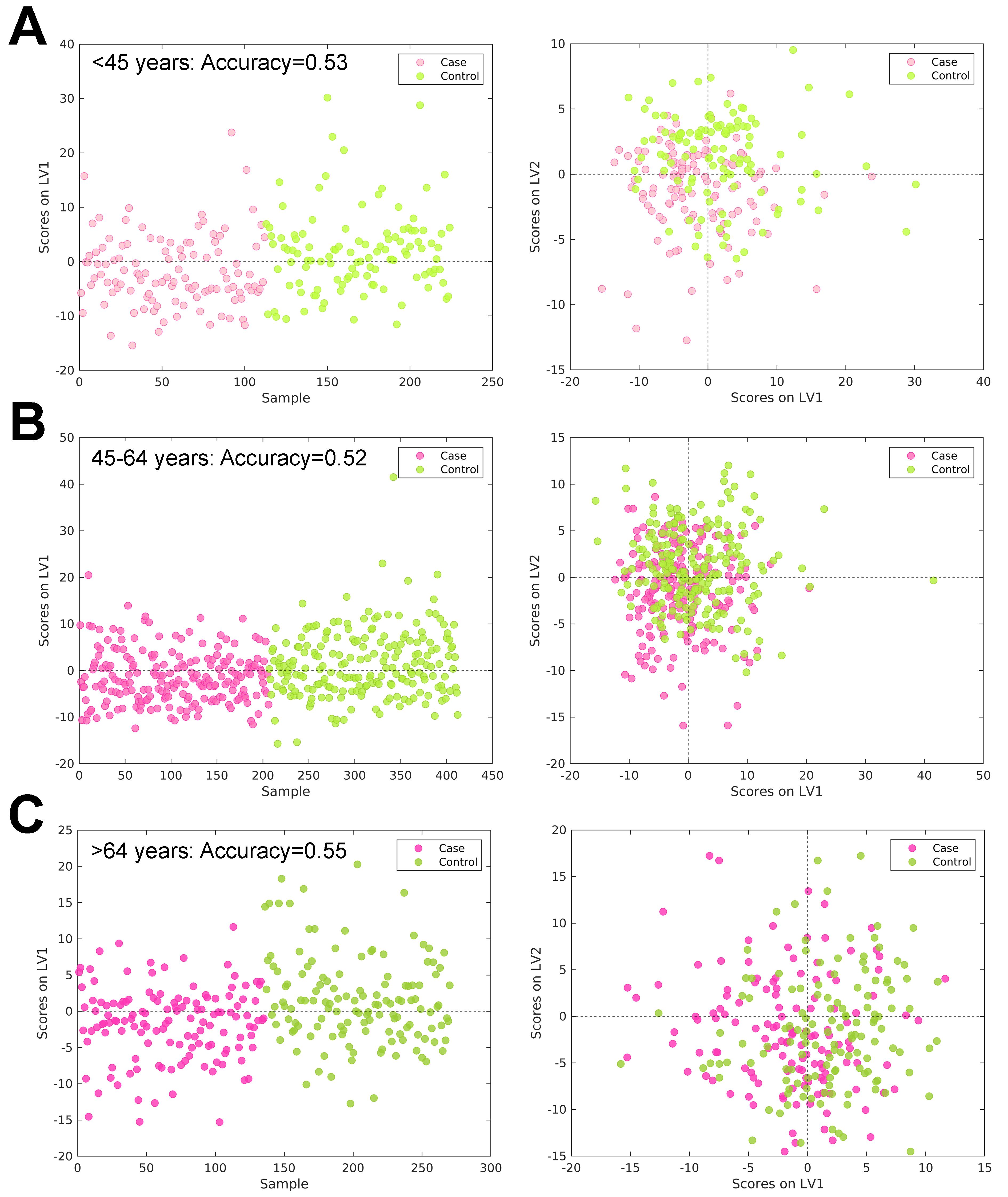

Supplement: Supplementary file 3 [file Image_3.jpeg]

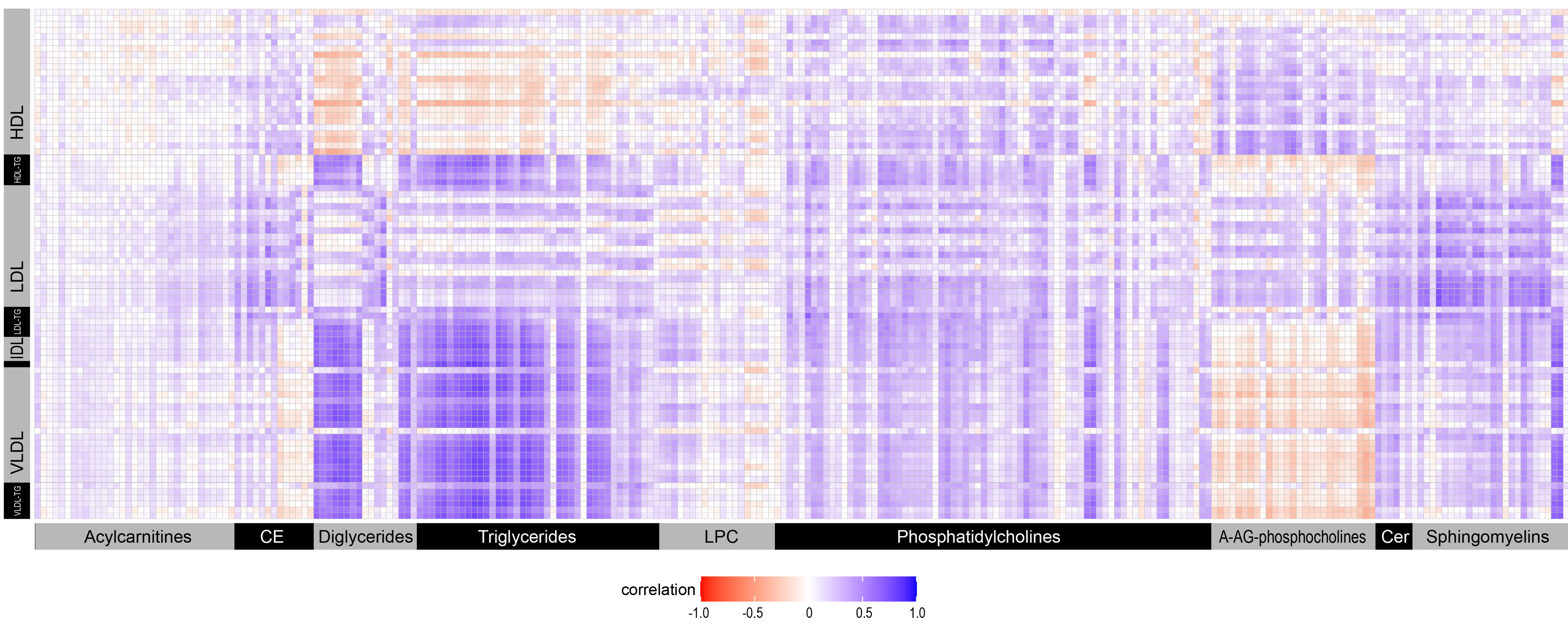

Supplement: Supplementary file 4 [file Image_4.jpeg]

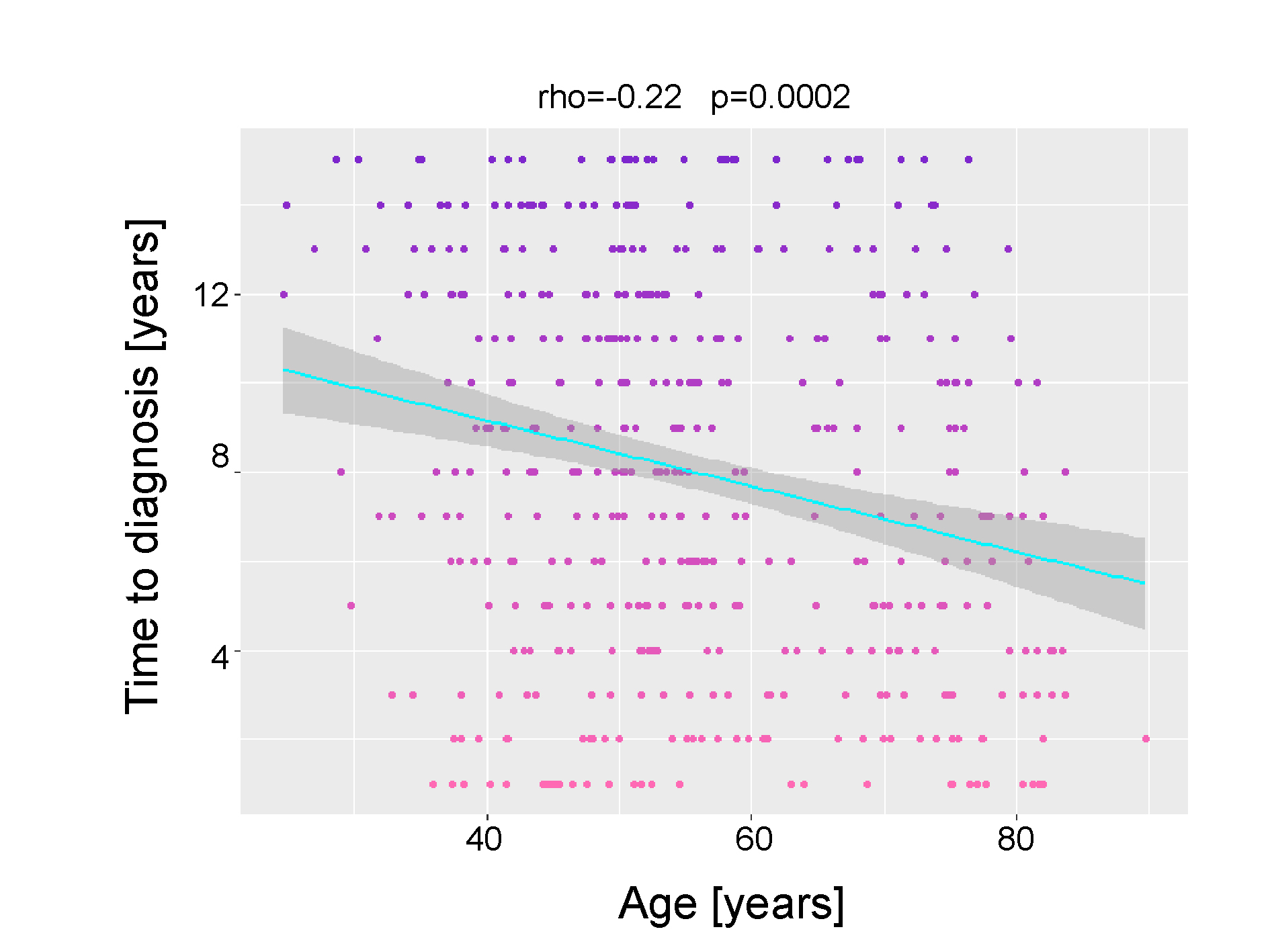

Supplement: Supplementary file 5 [file Image_5.jpeg]
